# Bioaccessibility of Rosmarinic Acid and Basil (*Ocimum basilicum* L.) Co-Compounds in a Simulated Digestion Model—The Influence of the Endogenous Plant Matrix, Dose of Administration and Physicochemical and Biochemical Digestion Environment

**DOI:** 10.3390/molecules29040901

**Published:** 2024-02-18

**Authors:** Łukasz Sęczyk, Barbara Kołodziej

**Affiliations:** Department of Industrial and Medicinal Plants, University of Life Sciences in Lublin, 15 Akademicka Str., 20-950 Lublin, Poland; barbara.kolodziej@up.lublin.pl

**Keywords:** antioxidants, bioaccessibility, dry extract, food matrix, in vitro digestion, natural products, nutraceuticals, polyphenols, rosmarinic acid

## Abstract

The objective of this study is to determine the effect of endogenous plant matrix components, dose and digestion-related factors on the bioaccessibility of rosmarinic acid and basil co-compounds in in vitro digestion conditions. Different forms of administration, i.e., basil raw plant material, dry extract, and isolated rosmarinic acid at various doses, were applied for the digestion experiment. To evaluate the contribution of biochemical and physicochemical digestion factors, samples were subjected to a full digestion process or treated only with a digestion fluid electrolyte composition without using biochemical components (i.e., digestion enzymes and bile salts), and bioaccessibility was monitored at the gastric and intestinal steps of digestion. The results showed that the components of the endogenous raw plant matrix significantly limited the bioaccessibility of rosmarinic acid and basil co-compounds, especially at the gastric stage of digestion. Physicochemical digestion factors were mainly responsible for the bioaccessibility of basil phytochemicals. Higher doses allowed maintenance of bioaccessibility at a relatively similar level, whereas the most negative changes in bioaccessibility were induced by the lowest doses. In conclusion, the determination of the bioaccessibility of bioactive phytochemicals from basil and factors influencing bioaccessibility may help in better prediction of the pro-health potential of this plant.

## 1. Introduction

Basil (*Ocimum basilicum* L.), sometimes called the “king of herbs” due to the origin of the name *Basileus* (gr.) meaning king or emperor, is one of the most popular culinary herbs worldwide [1,2]. It is highly appreciated for its unique taste and aroma; therefore, it is commonly used as seasoning for various foods and dishes, e.g., pizza, pasta, salads, or soups [3,4]. Furthermore, basil has a long tradition of use in folk medicine, especially in Ayurvedic and Unani phytotherapeutic systems, as a remedy for pain and disorders of the digestive, urinary, upper respiratory, cardiovascular, and nervous systems [2,3,5].

Scientific evidence from recent years indicates that the health-promoting potential of basil is associated with the high abundance of its bioactive compounds, mainly polyphenols and essential oils [5,6,7]. One of the prevailing non-volatile bioactive phytochemical constituents of basil, as well as other aromatic and medicinal plants of the mint (*Lamiaceae*) family, is rosmarinic acid [8,9,10]. Rosmarinic acid was isolated for the first time more than 65 years ago by Italian chemists Scarpati and Oriente from rosemary (*Rosmarinus officinalis* L.) [11,12,13]. Structurally, it is an ester, specifically, a depside of caffeic acid and 3,4-dihydroxy phenyllactic acid, which is biosynthesized in plants in the phenylpropanoid pathway from aromatic amino acid precursors L-phenylalanine and L-tyrosine [13,14,15]. In recent years, this compound has gained particular attention from the scientific community as a bioactive substance with multidirectional properties, including antidiabetic, antimicrobial, antioxidant, antiallergic, and cytoprotective (cardioprotective, hepatoprotective, nephroprotective, and neuroprotective) activity, and is regarded as a promising therapeutic agent reducing the risk and development of some metabolic disorders and civilization diseases [13,14,15,16,17,18].

Nevertheless, despite the well-documented in vitro and ex vivo (e.g., cell line studies) potential of rosmarinic acid, the final advantageous effects in vivo can be limited by its low bioaccessibility and the resulting bioavailability and bioactivity [17,19]. Bioaccessibility characterizes the part of an ingested substance released from the food matrix during digestion and available for absorption, whereas bioavailability is regarded as the part of an ingested substance that is consecutively released, absorbed, and delivered to tissues, where it can exert biological functions (which is referred to as bioactivity) [19,20,21].

During the digestion process, many complex factors can affect the digested material and bioactive compounds. They can be divided into physicochemical factors (temperature, pH, and interactions with electrolyte components) and biochemical factors (the action of digestion enzymes and bile salts) [22,23,24]. In specific conditions of the digestion system, phytochemicals, including phenolic substances, undergo various transformations that influence their bioaccessibility and further activity. Many reports show that degradation or conversion of polyphenols to derivatives as well as complexation with macromolecular food matrix components can restrict desired bioaccessibility by decreasing their occurrence in a native and absorption-susceptible form [20,25,26].

Therefore, to overcome the limitations in the bioaccessibility and subsequent bioavailability and bioactivity of rosmarinic acid and other phytochemicals, various strategies are being developed, for example, the modification and optimization of the formulation, dosage, and routes of administration and delivery [17,27].

New knowledge about the factors influencing the bioaccessibility of rosmarinic acid and other phytochemicals from natural sources may contribute to revealing its favorable properties in vivo and reinforce its application in phytotherapy. Given the foregoing, the objective of this study is to determine the effects of the dose and form of application (isolated rosmarinic acid, basil dry extract, plant material) as well as the contribution of physicochemical and biochemical factors present during artificial digestion on the potential bioaccessibility of rosmarinic acid. Furthermore, the influence of digestion conditions on the total phenolic content and in vitro antioxidant activities of plant material and dry extract was evaluated.

Comparisons of various forms of application enabled the estimation of the role of endogenous plant matrix components (co-extractable compounds in dry extracts and a complex matrix in plant material) in the bioaccessibility of phytochemicals. The digestion experiment design included the application of parallel procedures without and with the use of biochemical components of digestion fluids (enzymes and bile salts) to demonstrate the impact of physiochemical factors and biochemical factors, respectively.

To the best of our knowledge, this is the first report comprehensively describing the bioaccessibility of rosmarinic acid and other compounds from basil affected by dose-, food matrix-, and digestion-related factors.

## 2. Results

The characterization of the plant material and dry extract, including the content of phytochemicals, their stability during drying, and extract yield, is presented in Table 1. The negative effect of extract drying on the rosmarinic acid content, total phenolic content, and antioxidant properties was very weak and statistically insignificant, indicating the very high stability of basil phytochemicals during solvent removal using rotary evaporation.

**Figure 1:**
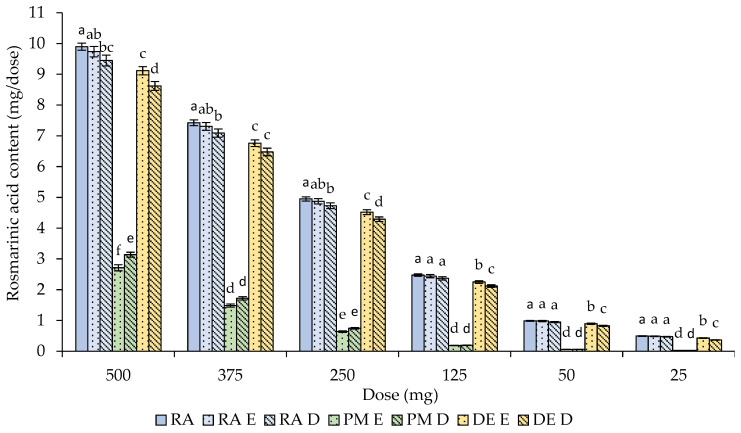
Effect of the simulated gastric digestion on the rosmarinic acid content. Bars represent means ± SD. Means indicated by different lowercase letters within the same dose differ significantly (*p* < 0.05). Abbreviations: RA—rosmarinic acid; PM—plant material; DE—dry extract; E—samples after electrolyte treatment (action of physicochemical factors); D—digested samples (action of physicochemical and biochemical factors). Dose represents the dose of applied plant material or corresponding doses of dry extract and rosmarinic acid. RA sample represents the rosmarinic acid content of untreated (before digestion) plant material.

The effects of the in vitro gastric digestion on the rosmarinic acid content and corresponding percentage bioaccessibility are shown in Figure 1 and Table 2, respectively.

After gastric digestion, the highest contents of rosmarinic acid, reflected by the highest bioaccessibilities, were observed after the application of the isolated compound alone. The treatment only with the physicochemical factors (RA E), regardless of the dose, had no significant influence on the rosmarinic acid content compared to its initially dosed amount (RA), which resulted in percentage bioaccessibilities close to 100%. In the case of the full digestion process, including the action of the biochemical factors, i.e., digestion enzymes and bile salts (RA D), slightly lower but also very high bioaccessibilities (about 95%) at all the tested dose ranges were obtained (without a statistically significant difference compared to the physicochemical treatment—RA E). Slightly lower but also relatively high bioaccessibilities were observed after the digestion of the dry material, where the bioaccessibility ranged from 87.5% to 92.3% in the DE E samples and from approximately 74.8% to 87.5% in the DE D, samples depending on the dose. The lowest bioaccessibilities were obtained at the lowest doses (25 mg); however, a statistically significant difference compared to the other doses was observed only in the DE D sample.

The most prominent effect of the dose and the lowest bioaccessibility compared to the other forms of rosmarinic acid application at the gastric step of digestion were observed in the plant material variant. The bioaccessibility of rosmarinic acid from the plant material progressively decreased along with the decreasing dose and ranged from approximately 5.5% to 27.4% in the PM E samples and from 5.6% to 31.7% in the PM D samples. In general, there were no statistically significant differences between the application of the physicochemical and biochemical factors in the same variant of digested material.

**Figure 2:**
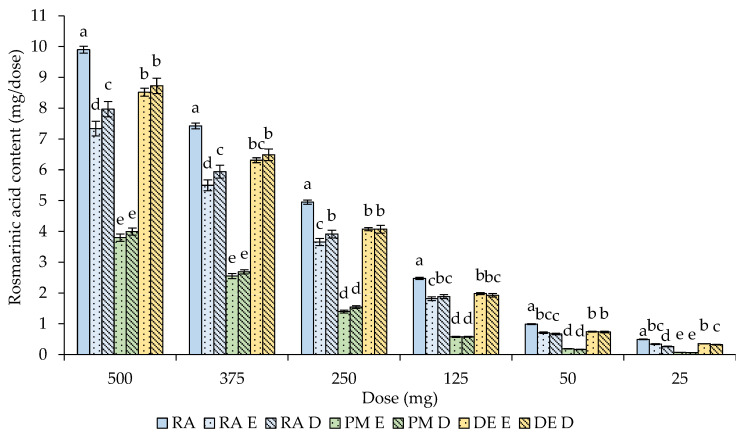
Effect of the simulated gastrointestinal digestion on the rosmarinic acid content. Description as in Figure 1.

The effects of the in vitro gastrointestinal digestion on the rosmarinic acid content and corresponding percentage bioaccessibility are shown in Figure 2 and Table 3, respectively.

Interestingly, at the intestinal digestion step, the highest bioaccessibility of rosmarinic acid was determined in the dry extracts; however, it decreased with the decreasing application dose. It ranged from 72.0% to 86.3% and from 65.9% to 88.4% in the DE E and DE D samples, respectively, without statistically significant differences between the samples at the corresponding doses. Slightly lower bioaccessibilities were found in the case of rosmarinic acid applied as an isolated compound, i.e., from 69.3% to 74.1% in RA E and from 53.4% to 80.5% in RA D, and the bioaccessibility decreased along with the decreasing dose. Similarly to the gastric step of digestion, the lowest bioaccessibilities were obtained in the plant material. In this case, the dose effect was also the most prominent. The bioaccessibility successively decreased with the decreasing dose. It ranged from 14.7% to 38.4% in PM E and from 13.0% to 40.4% in PM D. Only a slight and, in the vast majority of cases, insignificant influence on the contribution of the biochemical factors in the same variant of digested material was found.

Furthermore, differences in the bioaccessibility of rosmarinic acid between the digestion steps were found, e.g., the bioaccessibilities of rosmarinic acid applied as an isolated compound were significantly higher after the gastric digestion compared to the intestinal digestion, and, conversely, higher bioaccessibilities in the plant material variant were determined after the intestinal digestion.

Changes in the phytochemical contents and bioaccessibility of the dry extracts and plant material in the simulated digestion conditions reflected by the total phenolic content are shown in Table 4.

Similar to the results for rosmarinic acid, higher bioaccessibilities were obtained in the dry extracts than in the plant material, and the most pronounced negative dose effect was observed at the lowest doses (i.e., 25 and 50 mg). In turn, at higher doses, especially in the range of 250–500 mg, the bioaccessibility was maintained at a relatively similar level, both at the gastric and intestinal digestion steps. After the gastric digestion, depending on the dose, the bioaccessibility in the dry extracts ranged from 62.0% to 95.3% in samples treated with electrolytes (DE E) and from 58.4% to 95.4% in digested samples (DE D), whereas in the plant material variant, it ranged from 49.3% to 68.3% and from 25.7% to 70.4% in the PM E and PM D samples, respectively. After the intestinal digestion, the bioaccessibility of the dry extract ranged from 35.0% to 92.3% in the DE E samples and from 28.8% to 89.8% in the DE D samples. In the plant material variant, it ranged from 40.2% to 77.4% and from 34.8% to 71.8% in the PM E and PM D samples, respectively. In general, at the higher doses (especially in the range of 250–500 mg), there were no statistically significant differences between the samples after the electrolyte treatment and the full digestion process.

The effects of artificial digestion on the antioxidant activity expressed by the ability to reduce Fe^3+^ and neutralize ABTS^•+^ are presented in Table 5 and Table 6, respectively.

In accordance with the aforementioned findings, in most cases, the bioaccessibility was relatively similar when the higher doses (250–500 mg) were applied, and the most prominent decrease was observed after the application of the lowest doses (25 and 50 mg).

In the case of the Fe^3+^ reducing power analysis (Table 5), the antioxidative bioaccessibility at the gastric digestion step ranged from 60.4% to 78.4% after the electrolyte treatment of the dry extract (DE E), from 61.5% to 78.8% in the digested samples (DE D), and from 37.9% to 51.5% and 35.2% to 63.5% for PM E and PM D (plant material samples), respectively. At the intestinal digestion step of the dry extract, it ranged from 32.5% to 67.9% in the electrolyte treatment variant (DE E) and from 33.1% to 61.9% in the digested samples (DE D). In the plant material samples, it ranged from 27.7% to 85.4% and from 20.7% to 90.0% in PM E and PM D, respectively. In the case of the higher doses (250–500 mg), a significant difference between samples subjected to the electrolyte treatment and digested samples was observed in the variant with plant material after the gastric digestion, where PM D > PM E. In other cases, the differences were relatively small and mostly statistically insignificant. Furthermore, both at the gastric and intestinal steps of digestion, significantly higher bioaccessibilities were found in the dry extracts.

In the case of antiradical activity against ABTS^•+^ (Table 6), the bioaccessibility of the dry extract at the gastric digestion step was significantly higher compared to that of the plant material—it ranged from 65.0% to 79.5% in the DE E samples and from 50.3% to 68.6% in the DE D samples; in the plant material, it ranged from 35.2% to 43.9% and from 35.5% to 50.2% in PM E and PM D, respectively. Significant differences, even at the higher doses, were found between samples subjected to the electrolyte treatment and application of biochemical factors—DE E > DE D and PM E < PM D. Unexpectedly, at the intestinal step of digestion, relatively similar bioaccessibilities were found in the dry extracts and plant material. They ranged from 40.5% to 65.0% in DE E and from 35.4% to 69.3% in DE D in the dry extract variant, and from 46.3% to 72.6% and 31.7% to 67.3% in PM E and PM D, respectively, in the plant material samples. The electrolyte treatment in the plant material samples resulted in higher bioaccessibilities compared to the full digestion process, whereas this effect in the dry extracts varied among the doses.

## 3. Discussion

Bioaccessibility can be regarded as a consequence of two main phenomena influencing the content of phytochemicals in the potentially bioaccessible fraction: stability of compounds (i.e., resistance to degradation) and occurrence in an unbound form (mainly with macromolecular compounds) predisposing for absorption [25,28,29]. Taking into account the chemical features of polyphenols, including rosmarinic acid, it can be assumed that their stability during in vitro digestion can be mainly attributed to the action of physicochemical factors, e.g., temperature, pH, and electrolytes, whereas complexation with food matrix compounds and digestive enzymes can influence their “free” level, giving final bioaccessibility [17,30].

In this study, a comparison of the results from the electrolyte treatment in analogical experimental conditions without the use of digestion enzymes and bile salts with those obtained in the full digestion procedure (with digestion enzymes and bile salts) was performed to evaluate the contribution of the physicochemical and biochemical digestion environment on bioaccessibility. The results show that the bioaccessibility influenced by the physicochemical and biochemical factors was at a relatively similar level (especially at the higher doses). This observation indicates that the physicochemical environment was mainly responsible for the bioaccessibility of basil compounds, and the presence of biochemical factors (digestion enzymes and bile salts) did not strongly modify the bioaccessibility of basil phytochemicals.

It has been reported that, on the one hand, the action of biochemical factors can facilitate compound release from the physical food matrix structure and complexes with its macromolecular components; on the other hand, complexation with digestion enzymes may decrease the content of free compounds [25,31,32]. Presumably, the limited effect of biochemical factors on bioaccessibility found in this study was connected with the specific character of the applied phytochemicals subjected to digestion. Even raw plant material, i.e., dried basil leaves, is relatively low in digestion-susceptible proteins, starch, and lipids, along with a high share of indigestible compounds, mainly dietary fiber. Despite different types and characteristics of digested material, some similar trends in the contribution of physiochemical and biochemical factors were shown in other studies, where a similar study model was applied [33,34].

Our results indicate that the form of application of phytochemicals strongly influences their bioaccessibility. The experiment model, in which corresponding amounts of phytochemicals in various forms of application were used, taking into account the high stability of the compounds in the dry extract (Table 1), allows speculating about the role of endogenous food matrix components, both in solid state and co-extractable, on bioaccessibility. The application of raw plant material allows the delivery of all phytochemicals contained in the material to the digestion system, compared to only extractable and post-processing stable constituents in the case of plant extracts and isolated compounds. Nevertheless, other food matrix components delivered with phytochemicals can retain bioactive substances, decreasing their extractability and availability in the free form during digestion. Similarly to this study, the negative effects of raw plant material application for digestion (vs. dry extracts) on in vitro bioaccessibility were observed in our previous study on *Rhodiola rosea* compounds [35]. Furthermore, another model study on phenolic standards shows that exogenic food matrices, in particular those rich in dietary fiber, can strongly limit the bioaccessibility of phenolic compounds [26].

Phenolic–food matrix interactions at the molecular level can be considered as the ability to form associates with food matrix macromolecular components, i.e., proteins, fats, and dietary fiber [36,37,38]. The most common complexation has a reversible character (stabilized by weak non-covalent bonds); however, in the case of proteins, under certain circumstances (i.e., after phenolic oxidation to reactive quinones), irreversible covalent interactions are also possible [32,38].

Aside from the aforementioned negative effects on the concentration of compounds available for absorption, it has been reported that reversible interaction can allow the delivery of ingested substances to lower parts of the digestion tract [25,38]. This effect was also found in this study, where higher bioaccessibility of rosmarinic acid from the plant material was obtained after the intestinal digestion than after the gastric digestion (Table 2 and Table 3). Given the relatively weak effect of the biochemical factors, it may be speculated that rosmarinic acid bound during the gastric digestion process was released in the intestinal digestion step due to the changes in the physicochemical digestion environment rather than the potential food matrix decomposition progressing during the subsequent digestion phases.

It is reported that acidic conditions (like at the gastric phase) allow higher stability of phenolic compounds, whereas in a neutral (like at the intestinal phase) and, especially, an alkaline environment, degradation is much more facilitated [39,40]. Conversely, some studies have suggested that phenolic compounds at lower pH are characterized by a higher affinity for dietary fiber fractions [41,42]. Probably, such phenomena also contributed to the results obtained in this study, where despite the very high stability of pure rosmarinic acid in gastric conditions, its bioaccessibility when it was applied in the raw plant material was significantly lower (Table 2), whereas the intestinal conditions caused lower stability of this compound (in its pure form) but allowed its higher release from the matrix (Table 3). Gayoso et al. showed a similar tendency, i.e., very high stability and bioaccessibility of isolated rosmarinic acid in gastric conditions (around 92%) and its significant decrease in intestinal conditions; however, the food matrix effects were not investigated [30].

Furthermore, our results show that the administration of rosmarinic acid as a complex mixture, i.e., with co-extractable compounds in the dry extract form, in comparison with isolated compounds, was an important factor modifying its bioaccessibility. In addition to interactions with macromolecular plant matrix components, the phenomenon of interactions with low-molecular substances is indicated in the literature reports. It has been shown that synergistic or antagonistic interactions between phenolic molecules can influence the stability and properties of mixtures (including their antioxidant status), which may consequently affect their potential bioaccessibility [39,43,44]. In this study, the favorable effect of the co-compounds was found at the intestinal digestion stage, and the bioaccessibility of rosmarinic acid was higher when applied as a dry extract than the isolated compound alone (Table 3).

Another factor affecting the bioaccessibility of basil phytochemicals was the dose of administration. Interestingly, it was found that the application of digested materials in higher doses enabled the maintenance of the bioaccessibility at a relatively similar level, whereas the most significant negative changes were observed at the lowest doses (25–50 mg). Probably, this effect may be attributed to the lower ratio of phytochemicals introduced to the artificial digestion system versus its constant constituent composition, which resulted in a higher negative response of phytochemicals on the digestion environment and, consequently, caused more prominent negative changes in bioaccessibility.

The administration of rosmarinic acid and other bioactive phytochemicals at appropriate doses that ensure sufficiently high bioaccessibility can allow obtaining their effective concentration in vivo to exert desired therapeutic effects. Furthermore, although naturally occurring compounds, especially those derived from edible plants, are generally regarded as safe, the dose-dependent effects influencing bioaccessibility should be regraded during the development and optimization of the dose of phytopharmaceutical preparations to provide pro-health properties without potential side effects [27,28,45].

The literature data show that, besides the specific features, including the type, structure, and chemical properties and the concentration of molecules, the physiochemical conditions of the surrounding medium, i.e., temperature, pH, electrolyte composition, and presence of other low-molecular co-compounds, affect the stability, activity, and affinity of phenolic compounds for the macromolecular components in the digestion system and the food matrix [25,37,39].

Hence, the bioaccessibility of basil phytochemicals is a result of many factors, which comprise both unmodifiable digestion-related factors (i.e., physicochemical and biochemical conditions at the subsequent steps of digestion) and modifiable factors, such as the dose and form of administration. This multitude of factors that are mostly difficult to predict provides a unique complex system of dependencies between digested material and digestion environment, influencing the variability of obtained results.

## 4. Materials and Methods

### 4.1. Chemicals and Reagents

Rosmarinic acid standard (≥98%); digestion fluids salts and reagents, including hog pancreas α-amylase; porcine pepsin; porcine pancreatin, bovine bile extracts, elution solvents—HPLC gradient-grade acetonitrile and formic acid; total phenolic and antioxidant tests reagents and standards—Folin and Ciocalteu′s phenol reagent, 3,4,5-Trihydroxybenzoic acid (Gallic acid), 2,2′-Azino-bis(3-ethylbenzothiazoline-6-sulfonic acid) diammonium salt (ABTS), 2,3,5-Triphenyltetrazolium chloride (TPTZ), and (±)-6-Hydroxy-2,5,7,8-tetramethylchromane-2-carboxylic acid (Trolox) were purchased from Sigma–Aldrich (St. Louis, MO, USA). All other chemicals were of analytical grade.

### 4.2. Plant Material

Basil (*Ocimum basilicum* L. *var.* ‘Bonazza’) plants were cultivated in eastern Poland (South Podlachian Lowland). Seeds were purchased from the Graines Voltz Company (Colmar, France). Leaves were collected from plants at about 90% flowering stage and dried at 40 °C in a laboratory convection drier (Binder GmbH, Tuttlingen, Germany). Then, dried plant material was milled into a fine powder (passing through a 1.2 mm square mesh) and stored in an aluminum foil-spouted ziplock bag before use.

### 4.3. Extraction Procedure

For the isolation of phytochemicals from the basil leaves, a three-step sequential extraction was applied using 80% (*v*/*v*) methanol (MeOH) as a solvent (Table 7).

The extraction was assisted by ultrasounds generated in a laboratory ultrasound water bath. After each extraction step, samples were centrifuged (7728× *g*, 10 min, 20 °C), and supernatants were separated. Separated supernatants were combined and filtered in the Buchner funnel through a cellulose filter using external pressure above the solution. This extraction procedure was applied for both the phytochemical analysis of the plant material and the preparation of dry extracts (Section 4.4).

### 4.4. Dry Extract Preparation

To obtain dry extracts, the solvent was evaporated under reduced pressure in a rotary evaporator (INGOS, Prague, Czech Republic). The temperature of the water bath was set at 50 °C. The pressure was manually adjusted based on visual monitoring of foaming in the evaporating flask. The drying was completed within 2 h. After processing, the crude extract weight was determined using a laboratory scale, and the extract yield and plant material/extract ratio were calculated. Before use, the dry extract was manually pulverized to obtain homogeneous powder (passing through a 1.2 mm square mesh).

#### Stability of Phytochemicals in the Dry Extract

For the determination of the stability of basil compounds during dry extract preparation, an amount of the dry extract corresponding to the plant material (determined based on the extract yield—Table 1) was re-dissolved in 80% MeOH for 10 min using an ultrasound water bath set at 40 °C. The reconstituted extract was subjected to analysis of phytochemicals (Section 4.6), and the obtained results were compared to those for plant material extracts (before drying). The stability was expressed as a percentage share of phytochemical contents or antioxidant activity in the re-dissolved dry extract with regard to contents corresponding to the plant material extract (content or activity before drying/content or activity after drying ×100%).

### 4.5. Artificial Digestion and Determination of Potential Bioaccessibility

#### 4.5.1. Experiment Design and Preparation of Samples

To evaluate the effect of the dose of administration, the plant material and dry extracts were subjected to digestion in various amounts. During the digestion experiment, the doses of whole plant material (dried leaf powder) were established at 500, 375, 250, 125, 50, and 25 mg. The corresponding doses of the dry extract and rosmarinic acid were calculated based on the extract yield and the determined rosmarinic acid content (Table 1). Specifically, the doses of the dry extract were 153.4, 115.0, 76.7, 38.3, 15.3, and 7.7 mg. Rosmarinic acid was applied in amounts of 9.90, 7.42, 4.95, 2.47, 0.99, and 0.49 mg regarding the corresponding doses of the plant material and 9.87, 7.40, 4.94, 2.47, 0.99, and 0.49 mg regarding the corresponding doses of the dry extracts (taking into account the stability of this compound).

The application of various forms of rosmarinic acid, i.e., in the plant material, dry extracts, and as an isolated compound at corresponding doses, was assumed to estimate the effect of the co-extractable compounds (in the dry extracts) and the complex plant matrix (in the plant material) on its bioaccessibility. All variants of samples were subjected to the action of only physicochemical digestion factors (treatment with electrolytes without using digestion enzymes and bile salts) or a complete digestion process was performed (using digestion enzymes and bile salts) to estimate the contribution of the physicochemical and biochemical digestion factors. Bioaccessibility was monitored at the two main steps of digestion—gastric and intestinal digestion.

The INFOGEST standardized static model of artificial digestion based on the international consensus described by [24] and developed by [46] was used for the determination of potential bioaccessibility. Due to the general recommendations of the protocol of artificial digestion [46], which is originally intended for well-hydrated food products (in contrast to high-solid materials containing a residual amount of water, such as powdered materials), and the requirements of material preparation for digestion that reflects consumption assumptions (e.g., herbs are consumed in lower quantities compared to staple foods), the sample size was reduced 10 times before the digestion process, i.e., instead of 5 g of naturally water containing food (as recommended for such type products by the procedure), 0.5 g of powdered plant material was used or an equivalent of the dry extract/rosmarinic acid standard and the procedure was unified for all doses.

#### 4.5.2. Digestion Procedure

The main INFOGEST protocol digestion parameters, including the composition and concentration of electrolytes, enzymes, bile salts, time and temperature conditions, and other general recommendations, were implemented from the aforementioned protocol [46].

In brief, for the simulation of the oral phase, the samples were combined with 5 mL of artificial saliva composed of artificial saliva electrolytes (pH 7) and alpha-amylase at a concentration of 75 U/mL and incubated and mixed for 2 min at 37 °C without light. After the oral phase, the samples were mixed with 10 mL of artificial gastric fluid containing gastric fluid electrolytes (pH 3), pepsin (2000 U/mL), and gastric lipase (60 U/mL) and then incubated with mixing for 120 min at 37 °C without light for simulation of gastric digestion. At the intestinal phase of digestion, gastric chyme was mixed with 20 mL of artificial intestinal juice containing intestinal juice electrolytes (pH 7), pancreatin (100 U/mL—regarding tryptic activity), and bile extract (10 mM bile salts). The samples were incubated with mixing for 120 min at 37 °C without light.

After completing the digestion process, in order to receive an in vitro bioaccessible fraction, the samples were centrifuged, and the collected supernatants were ultrafiltered using a centrifuge tube filter equipped with a 5 kDa membrane [35].

Parallelly, samples without digestion enzymes and bile salts were prepared. For the preparation of blank samples for spectrophotometric analyses, an analogical procedure was performed without using digested material.

#### 4.5.3. Bioaccessibility Calculation

In vitro bioaccessibility (BA) expressed as the percentage share of phytochemical content (PC) or antioxidant activity (AA) determined for a potentially bioaccessible fraction (after digestion—AD) in relation to the initial content (or antioxidant activity) evaluated for 80% MeOH extracts (BD—before digestion) was calculated using the following formula:

(1) $$BA\left(\%\right)=\frac{{PC(AA)}_{AD}}{{PC(AA)}_{BD}}\times 100\%$$

### 4.6. Phytochemical Analyses

#### 4.6.1. Determination of Rosmarinic Acid Content by High-Performance Liquid Chromatography (HPLC)

Chromatographic quantification of rosmarinic acid was performed on an Agilent 1260 Infinity II system (Agilent Technologies, Santa Clara, CA, USA). The system was equipped with a quaternary solvent delivery module, a column thermostat, an automated and thermostatted sample injector, and a photodiode array detector (DAD). The samples were separated on a Luna C18 (250 mm × 4.6 mm, 5 µm) analytical column (Phenomenex, Torrance, CA, USA). The injection volume was 10 µL, and the column thermostat was set at 20 °C. The eluents were 0.1% (*v*/*v*) formic acid in ultrapure water (A) and in acetonitrile (B) at the following gradient program: 0 min (B 5%), 1 min (B 5%), 4 min (B 25%), 17 min (B 25%), 20 min (B 100%), 23 min (B 100%), 26 min (B 5%), and 30 min (B 5%) maintained during separation at a constant flow rate (1 mL∙min^−1^). Chromatograms were recorded at 330 nm, and DAD spectra were monitored from 200 to 400 nm. Rosmarinic acid was identified by comparing the spectra and retention time with the standard and quantified using an external calibration curve.

#### 4.6.2. Spectrophotometric Determination of Total Phenolic Content and in Vitro Antioxidant Activity of Extracts and Plant Material

The Folin–Ciocalteu reagent assay was performed for the determination of total polyphenols [47]. The ABTS^•+^ [48] decolorization assay and the Fe^3+^-TPTZ reduction assay [49] were applied for the evaluation of in vitro antioxidant activity. The procedures were adapted to a 96-well microplate scale, and absorbances were recorded using an Epoch 2 microplate reader (BioTek Instruments, Winooski, VT, USA). In brief, for the determination of the total phenolic content, aliquots (10 µL) of extracts or potentially bioaccessible fractions were combined with 100 µL of H_2_O and 20 µL of Folin–Ciocalteu reagent (diluted 1:5 with H_2_O; *v*/*v*). The reaction mixture was incubated for 3 min, and then 100 µL of 10% Na_2_CO_3_ (*w*/*v*) was added. After 30 min of incubation, the absorbance was read at 765 nm. For the determination of ABTS^•+^ antiradical activity, aliquots (5 µL) of extracts or potentially bioaccessible fractions were mixed with 300 µL of ABTS^•+^ reagent. ABTS^•+^ was generated using K_2_S_2_O_8_ following the original procedure [48]. Absorbance was measured after 120 min of incubation at 734 nm. For the determination of ferric reducing antioxidant power, aliquots (4 µL) of extracts or potentially bioaccessible fractions were combined with 40 µL of FRAP reagent prepared strictly according to the original procedure [49] and 200 µL of H_2_O. Absorbance was measured after 30 min of incubation at 593 nm.

Gallic acid was used as a standard for the total phenolic content, and Trolox was used for antioxidant activity quantification.

### 4.7. Statistics

Experimental data were expressed as the mean ± SD (standard deviation). All experiments were performed in triplicate (n = 3). Statistical analysis was performed using ANOVA (one-way analysis of variance) to analyze the differences among groups, followed by Tukey’s HSD post hoc test. Differences at *p* < 0.05 were considered significant. Statistica 6.0 software (StatSoft, Krakow, Poland) was applied for computer data analysis.

## 5. Conclusions

In conclusion, our studies showed that physicochemical digestion factors were mainly responsible for the in vitro bioaccessibility of basil phytochemicals. As shown, it can be limited by the low dose and the components of the endogenous raw plant matrix. Furthermore, it has been demonstrated that the form of phytochemical administration can significantly affect the potential bioaccessibility, which seems to be important, especially considering the applications of herbal products in phytotherapy. The determination of the bioaccessibility of bioactive phytochemicals from basil, with special emphasis on rosmarinic acid, and the factors influencing the bioaccessibility may help in a better evaluation of the pro-health potential of this plant or, in a broader perspective, ensure enhanced bioaccessibility in vivo (e.g., via optimization of the dose and form of administration) to exert desired biological functions. Although in vitro studies on bioaccessibility can provide valuable information about the potential fate of phytochemicals in the gastrointestinal tract, the prospects should include large-scale in vivo comparative studies aimed at assessing to what extent the in vitro bioaccessibility can be reflected in bioavailability and bioactivity in vivo, with regard to the very complex nature of the physiological mechanisms engaged in the absorption, distribution, and utilization of plant compounds.

## Figures and Tables

**Table 1:** Phytochemical characterization of plant material and dry extract.

Sample	RAC (mg∙g^−1^ DW)	TPC (mg GAE∙g^−1^ DW)	FRAP (mg TE∙g^−1^ DW)	ABTS (mg TE∙g^−1^ DW)	Extract Yield (mg DE∙g^−1^ PM)
Plant material	19.80 ± 0.06	53.88 ± 1.40	102.35 ± 1.68	149.22 ± 3.78	-
Dry extract	64.37 ± 0.37	174.11 ± 4.65	327.08 ± 5.41	485.52 ± 9.95	306.7
Stability (%)	99.72 ± 0.85	99.12 ± 2.59	98.06 ± 3.23	99.83 ± 2.29	-

Abbreviations: RAC—rosmarinic acid content; TPC—total phenolic content; FRAP—ferric reducing antioxidant power; ABTS—ABTS^•+^ antiradical activity; DW—dry weight; GAE—gallic acid equivalents; TE—Trolox equivalents; DE—dry extract; PM—plant material.

**Table 2:** Potential bioaccessibility of rosmarinic acid after the simulated gastric digestion.

Sample /Dose	Gastric Bioaccessibility (%)					
	RA E	RA D	PM E	PM D	DE E	DE D
500	98.4 ± 2.82 ^aA^	95.5 ± 2.91 ^abA^	27.4 ± 1.07 ^dA^	31.7±0.91 ^dA^	92.3 ± 1.90 ^bcA^	87.4 ± 1.95 ^cA^
375	98.4 ± 2.93 ^aA^	95.5 ± 3.04 ^abA^	20.0 ± 0.74 ^dB^	23.2±0.83 ^dB^	91.3 ± 1.98 ^bcA^	87.5 ± 2.16 ^cA^
250	98.5 ± 3.12 ^aA^	95.6 ± 3.19 ^abA^	13.0 ± 0.54 ^dC^	15.1±0.55 ^dC^	91.6 ± 2.16 ^bcA^	87.0 ± 1.99 ^cA^
125	98.7 ± 3.60 ^aA^	95.7 ± 3.62 ^abA^	7.7 ± 0.29 ^dD^	7.9±0.28 ^dD^	91.2 ± 2.27 ^bcA^	86.0 ± 2.20 ^cA^
50	99.4 ± 3.68 ^aA^	95.9 ± 3.47 ^abA^	6.0 ± 0.28 ^dDE^	6.3±0.27 ^dDE^	90.6 ± 2.49 ^bcA^	83.9 ± 2.21 ^cA^
25	98.6 ± 4.30 ^aA^	95.7 ± 4.27 ^aA^	5.5 ± 0.32 ^dE^	5.6±0.34 ^dE^	87.5 ± 2.44 ^bA^	74.8 ± 2.06 ^cB^

Data represent means ± SD. Means indicated by different lowercase letters in rows and uppercase letters in columns differ significantly (*p* < 0.05). Abbreviations: RA—rosmarinic acid; PM—plant material; DE—dry extract; E—samples after electrolyte treatment (action of physicochemical factors); D—digested samples (action of physicochemical and biochemical factors). Dose represents the dose of applied plant material or corresponding doses of dry extract and rosmarinic acid.

**Table 3:** Potential bioaccessibility of rosmarinic acid after the simulated gastrointestinal digestion.

Sample /Dose	Gastrointestinal Bioaccessibility (%)					
	RA E	RA D	PM E	PM D	DE E	DE D
500	74.1 ± 3.27 ^cA^	80.5 ± 3.44 ^bcA^	38.4 ± 1.31 ^dA^	40.4 ± 1.26 ^dA^	86.3 ± 1.79 ^abA^	88.4 ± 3.03 ^aA^
375	74.1 ± 3.33 ^cA^	80.0 ± 3.84 ^bcA^	34.3 ± 1.20 ^dB^	36.2 ± 1.03 ^dB^	85.2 ± 1.56 ^abA^	87.6 ± 3.03 ^aA^
250	73.9 ± 3.35 ^bA^	79.1 ± 3.63 ^abA^	28.2 ± 1.07 ^cC^	31.2 ± 0.97 ^cC^	82.6 ± 1.47 ^aAB^	82.6 ± 3.03 ^aAB^
125	73.4 ± 3.63 ^aA^	76.2 ± 3.84 ^aAB^	23.3 ± 0.86 ^bD^	23.4 ± 0.63 ^bD^	80.4 ± 1.73 ^aB^	78.1 ± 2.79 ^aBC^
50	71.8 ± 3.72 ^abA^	67.7 ± 3.69 ^bB^	18.9 ± 0.78 ^cE^	17.0 ± 0.59 ^cE^	75.5 ± 1.84 ^aC^	74.3 ± 3.19 ^abC^
25	69.3 ± 4.24 ^abA^	53.5 ± 3.35 ^bC^	14.7 ± 0.71 ^cF^	13.0 ± 0.54 ^cF^	72.0 ± 1.14 ^aC^	65.9 ± 2.91 ^aD^

Description as in Table 2.

**Table 4:** Effect of simulated digestion on the total phenolic content and bioaccessibility of basil phytochemicals.

	Sample /Dose	Total Phenolic Content (mg GAE/Dose)						Bioaccessibility (%)			
		PM	PM E	PM D	DE	DE E	DE D	PM E	PM D	DE E	DE D
Gastric digestion	500	26.94 ± 0.70 ^a^	18.38 ± 0.25 ^c^	18.96 ± 0.29 ^c^	26.70 ± 0.71 ^a^	25.30 ± 0.39 ^b^	25.08 ± 0.50 ^b^	68.3 ± 2.69 ^bA^	70.4 ± 2.93 ^bA^	94.8 ± 3.98 ^aA^	94.0 ± 4.39 ^aA^
	375	20.20 ± 0.53 ^a^	13.61 ± 0.18 ^c^	13.39 ± 0.19 ^c^	20.03 ± 0.53 ^ab^	19.07 ± 0.28 ^b^	19.09 ± 0.23 ^b^	67.4 ± 2.64 ^bA^	66.3 ± 2.67 ^bA^	95.3 ± 3.95 ^aA^	95.4 ± 3.70 ^aA^
	250	13.47 ± 0.35 ^a^	8.83 ± 0.05 ^c^	8.80 ± 0.11 ^c^	13.35 ± 0.36 ^a^	12.29 ± 0.17 ^b^	12.68 ± 0.19 ^b^	65.6 ± 2.08 ^bA^	65.4 ± 2.55 ^bA^	92.1 ± 3.74 ^aA^	95.0 ± 3.97 ^aA^
	125	6.73 ± 0.18 ^a^	3.85 ± 0.05 ^c^	3.49 ± 0.05 ^d^	6.68 ± 0.18 ^a^	6.08 ± 0.09 ^b^	6.02 ± 0.12 ^b^	57.2 ± 2.24 ^bB^	51.9 ± 2.07 ^bB^	91.1 ± 3.80 ^aA^	90.3 ± 4.18 ^aA^
	50	2.69 ± 0.07 ^a^	1.42 ± 0.04 ^d^	1.04 ± 0.02 ^e^	2.67 ± 0.07 ^a^	2.26 ± 0.05 ^b^	2.09 ± 0.05 ^c^	52.6 ± 2.82 ^bBC^	38.7 ± 1.83 ^cC^	84.7 ± 4.25 ^aA^	78.3 ± 4.05 ^aB^
	25	1.35 ± 0.04 ^a^	0.66 ± 0.03 ^d^	0.35 ± 0.01 ^e^	1.34 ± 0.04 ^a^	0.83 ± 0.04 ^b^	0.78 ± 0.04 ^c^	49.3 ± 3.15 ^bC^	25.7 ± 1.54 ^cD^	62.1 ± 4.51 ^aB^	58.4 ± 4.31 ^aC^
Gastrointestinal digestion	500	26.94 ± 0.70 ^a^	20.84 ± 0.30 ^c^	19.32 ± 0.26 ^d^	26.70 ± 0.71 ^a^	24.64 ± 0.36 ^b^	23.97 ± 0.52 ^b^	77.4 ± 3.15 ^bA^	71.8 ± 2.83 ^bA^	92.3 ± 3.80 ^aA^	89.8 ± 4.33 ^aA^
	375	20.20 ± 0.53 ^a^	15.49 ± 0.20 ^c^	14.03 ± 0.19 ^d^	20.03 ± 0.53 ^a^	18.37 ± 0.32 ^b^	17.96 ± 0.42 ^b^	76.7 ± 3.00 ^bA^	69.5 ± 2.74 ^bAB^	91.8 ± 4.04 ^aA^	89.7 ± 4.51 ^aA^
	250	13.47 ± 0.35 ^a^	10.18 ± 0.13 ^c^	8.93 ± 0.12 ^d^	13.35 ± 0.36 ^a^	11.65 ± 0.13 ^b^	11.08 ± 0.24 ^c^	75.6 ± 2.96 ^bA^	66.4 ± 2.60 ^cAB^	87.3 ± 3.30 ^abA^	83.1 ± 4.03 ^bAB^
	125	6.73 ± 0.18 ^a^	4.94 ± 0.09 ^c^	4.22 ± 0.05 ^d^	6.68 ± 0.18 ^a^	5.16 ± 0.08 ^bc^	5.33 ± 0.08 ^c^	73.4 ± 3.22 ^aA^	62.8 ± 2.33 ^bB^	77.3 ± 3.20 ^aB^	79.9 ± 3.27 ^aB^
	50	2.69 ± 0.07 ^a^	1.53 ± 0.03 ^b^	1.34 ± 0.02 ^c^	2.67 ± 0.07 ^a^	1.44 ± 0.04 ^bc^	1.36 ± 0.03 ^c^	56.8 ± 2.65 ^aB^	49.9 ± 2.09 ^bC^	54.0 ± 3.07 ^abC^	51.1 ± 2.41 ^bC^
	25	1.35 ± 0.04 ^a^	0.54 ± 0.03 ^b^	0.47 ± 0.02 ^b^	1.34 ± 0.04 ^a^	0.47 ± 0.02 ^b^	0.38 ± 0.02 ^c^	40.2 ± 3.46 ^aC^	34.8 ± 2.36 ^abD^	35.0 ± 2.41 ^abD^	28.8 ± 1.94 ^bD^

Data in the table represent means ± SD. Means indicated by different lowercase letters in rows for the content and means indicated by different lowercase letters in rows and uppercase letters in columns (separately for gastric and gastrointestinal digestion) for bioaccessibility differ significantly (*p* < 0.05). Abbreviations: PM—plant material; DE—dry extract; E—samples after electrolyte treatment (action of physicochemical factors); D—digested samples (action of physicochemical and biochemical factors). Dose represents the dose of applied plant material or corresponding doses of dry extract and rosmarinic acid. PM and DE samples represent the total phenolic content for untreated (before digestion) plant material and dry extract, respectively.

**Table 5:** Effect of simulated digestion on the ferric reducing antioxidant power and antioxidative bioaccessibility of basil phytochemicals.

	Sample /Dose	Ferric Reducing Antioxidant Power (mg TE/Dose)						Bioaccessibility (%)			
		PM	PM E	PM D	DE	DE E	DE D	PM E	PM D	DE E	DE D
Gastric digestion	500	51.18 ± 0.84 ^a^	26.37 ± 0.27 ^d^	32.50 ± 0.43 ^c^	50.16 ± 0.83 ^a^	39.32 ± 0.50 ^b^	39.53 ± 1.46 ^b^	51.5 ± 1.36 ^cA^	63.5 ± 1.88 ^bA^	78.4 ± 2.30 ^aA^	78.8 ± 4.22 ^aA^
	375	38.38 ± 0.63 ^a^	18.83 ± 0.31 ^d^	23.55 ± 0.33 ^c^	37.62 ± 0.62 ^a^	29.17 ± 0.55 ^b^	29.65 ± 1.04 ^b^	49.1 ± 1.60 ^cAB^	61.4 ± 1.87 ^bA^	77.6 ± 2.74 ^aA^	78.9 ± 4.06 ^aA^
	250	25.59 ± 0.42 ^a^	11.98 ± 0.18 ^e^	15.26 ± 0.17 ^d^	25.08 ± 0.42 ^a^	18.67 ± 0.23 ^b^	19.76 ± 0.22 ^c^	46.9 ± 1.48 ^cAB^	59.7 ± 1.64 ^bA^	74.4 ± 2.15 ^aAB^	78.8 ± 2.19 ^aA^
	125	12.79 ± 0.21 ^a^	5.43 ± 0.07 ^f^	6.56 ± 0.09 ^e^	12.54 ± 0.21 ^a^	9.24 ± 0.10 ^d^	9.94 ± 0.26 ^c^	42.4 ± 1.23 ^cBC^	51.3 ± 1.52 ^bB^	73.7 ± 2.03 ^aAB^	79.3 ± 3.39 ^aA^
	50	5.12 ± 0.08 ^a^	2.03 ± 0.07 ^c^	2.26 ± 0.04 ^c^	5.02 ± 0.08 ^a^	3.43 ± 0.08 ^b^	3.57 ± 0.14 ^b^	39.7 ± 2.01 ^bC^	44.2 ± 1.44 ^bC^	68.5 ± 2.75 ^aB^	71.1 ± 3.98 ^aA^
	25	2.56 ± 0.04 ^a^	0.97 ± 0.07 ^c^	0.90 ± 0.02 ^c^	2.51 ± 0.04 ^a^	1.51 ± 0.06 ^b^	1.54 ± 0.05 ^b^	37.9 ± 3.17 ^bC^	35.2 ± 1.43 ^bD^	60.4 ± 3.26 ^aC^	61.5 ± 2.91 ^aB^
Gastrointestinal digestion	500	51.18 ± 0.84 ^a^	34.73 ± 1.10 ^c^	31.66 ± 0.42 ^d^	50.16 ± 0.83 ^a^	42.80 ± 0.59 ^b^	45.11 ± 1.48 ^b^	67.9 ± 3.26 ^bA^	61.9 ± 1.83 ^bA^	85.4 ± 2.60 ^aA^	90.0 ± 4.44 ^aA^
	375	38.38 ± 0.63 ^a^	25.79 ± 0.30 ^d^	23.36 ± 0.28 ^e^	37.62 ± 0.62 ^a^	31.92 ± 0.47 ^c^	33.53 ± 0.91 ^b^	67.2 ± 1.89 ^bA^	60.9 ± 1.74 ^cA^	84.9 ± 2.66 ^aA^	89.2 ± 3.91 ^aA^
	250	25.59 ± 0.42 ^a^	16.19 ± 0.28 ^c^	15.03 ± 0.19 ^d^	25.08 ± 0.42 ^a^	21.09 ± 0.30 ^b^	21.70 ± 0.28 ^b^	63.3 ± 2.14 ^bAB^	58.8 ± 1.70 ^bA^	84.1 ± 2.58 ^aA^	86.6 ± 2.56 ^aA^
	125	12.79 ± 0.21 ^a^	7.73 ± 0.21 ^c^	6.44 ± 0.09 ^d^	12.54 ± 0.21 ^a^	9.23 ± 0.24 ^b^	9.22 ± 0.12 ^b^	60.4 ± 2.64 ^bB^	50.4 ± 1.51 ^cB^	73.6 ± 3.14 ^aB^	73.6 ± 2.16 ^aB^
	50	5.12 ± 0.08 ^a^	2.61 ± 0.07 ^c^	2.18 ± 0.04 ^d^	5.02 ± 0.08 ^a^	2.90 ± 0.08 ^b^	2.09 ± 0.16 ^c^	51.0 ± 2.20 ^bC^	42.7 ± 1.51 ^cC^	57.8 ± 2.56 ^aC^	41.6 ± 3.90 ^cC^
	25	2.56 ± 0.04 ^a^	0.83 ± 0.03 ^b^	0.85 ± 0.04 ^b^	2.51 ± 0.04 ^a^	0.69 ± 0.02 ^c^	0.52 ± 0.05 ^d^	32.5 ± 1.73 ^aD^	33.1 ± 1.94 ^aD^	27.7 ± 1.44 ^bD^	20.7 ± 2.49 ^cD^

Description as in Table 4.

**Table 6:** Effect of simulated digestion on the ABTS^•+^ antiradical activity and antioxidative bioaccessibility of basil phytochemicals.

	Sample /Dose	ABTS^•+^ Antiradical Activity (mg TE/Dose)						Bioaccessibility (%)			
		PM	PM E	PM D	DE	DE E	DE D	PM E	PM D	DE E	DE D
Gastric digestion	500	74.61 ± 1.89 ^a^	32.77 ± 0.33 ^e^	37.42 ± 0.48 ^d^	74.46 ± 1.53 ^a^	59.16 ± 0.83 ^b^	51.08 ± 1.06 ^c^	43.9 ± 1.55 ^dA^	50.2 ± 1.91 ^cA^	79.5 ± 2.74 ^aA^	68.6 ± 2.83 ^bA^
	375	55.96 ± 1.42 ^a^	24.49 ± 0.31 ^e^	27.97 ± 0.28 ^d^	55.85 ± 1.14 ^a^	44.22 ± 0.19 ^b^	37.81 ± 0.58 ^c^	43.8 ± 1.65 ^dA^	50.0 ± 1.77 ^cA^	79.2 ± 1.96 ^aA^	67.7 ± 2.42 ^bA^
	250	37.31 ± 0.94 ^a^	16.27 ± 0.18 ^e^	18.54 ± 0.22 ^d^	37.23 ± 0.76 ^a^	28.89 ± 0.30 ^b^	25.25 ± 0.57 ^c^	43.6 ± 1.58 ^dA^	49.7 ± 1.86 ^cA^	77.6 ± 2.40 ^aA^	67.8 ± 2.93 ^bA^
	125	18.65 ± 0.47 ^a^	7.65 ± 0.09 ^e^	8.87 ± 0.15 ^d^	18.62 ± 0.38 ^a^	15.05 ± 0.26 ^b^	12.69 ± 0.33 ^c^	41.1 ± 1.53 ^dAB^	47.6 ± 1.98 ^cAB^	80.9 ± 3.07 ^aA^	68.2 ± 3.16 ^bA^
	50	7.46 ± 0.19 ^a^	2.76 ± 0.06 ^e^	3.26 ± 0.06 ^d^	7.45 ± 0.15 ^a^	5.50 ± 0.11 ^b^	4.60 ± 0.13 ^c^	37.1 ± 1.78 ^dB^	43.7 ± 1.85 ^cB^	73.9 ± 2.93 ^aA^	61.9 ± 2.97 ^bA^
	25	3.73 ± 0.09 ^a^	1.31 ± 0.08 ^d^	1.32 ± 0.03 ^d^	3.72 ± 0.08 ^a^	2.42 ± 0.04 ^b^	1.87 ± 0.08 ^c^	35.2 ± 2.91 ^cB^	35.5 ± 1.83 ^cC^	65.0 ± 2.31 ^aB^	50.3 ± 3.12 ^bB^
Gastrointestinal digestion	500	74.61 ± 1.89 ^a^	54.13 ± 0.83 ^b^	50.16 ± 0.56 ^c^	74.46 ± 1.53 ^a^	48.41 ± 0.86 ^c^	51.57 ± 1.01 ^bc^	72.6 ± 2.95 ^aA^	67.3 ± 2.45 ^bA^	65.0 ± 2.49 ^bA^	69.3 ± 2.78 ^abA^
	375	55.96 ± 1.42 ^a^	40.17 ± 0.60 ^b^	36.53 ± 0.30 ^c^	55.85 ± 1.14 ^a^	35.97 ± 0.46 ^c^	38.12 ± 0.60 ^bc^	71.8 ± 2.88 ^aA^	65.3 ± 2.19 ^bAB^	64.4 ± 2.14 ^cA^	68.3 ± 2.48 ^abA^
	250	37.31 ± 0.94 ^a^	25.81 ± 0.43 ^b^	23.42 ± 0.21 ^c^	37.23 ± 0.76 ^a^	23.52 ± 0.37 ^c^	25.18 ± 0.46 ^b^	69.2 ± 2.90 ^aA^	62.8 ± 2.14 ^aAB^	63.2 ± 2.28 ^aAB^	67.7 ± 2.61 ^aAB^
	125	18.65 ± 0.47 ^a^	12.80 ± 0.18 ^b^	10.80 ± 0.09 ^c^	18.62 ± 0.38 ^a^	11.85 ± 0.53 ^bc^	11.38 ± 0.24 ^c^	68.7 ± 2.72 ^aA^	57.9 ± 1.95 ^bB^	63.7 ± 4.16 ^abA^	61.2 ± 2.52 ^abBC^
	50	7.46 ± 0.19 ^a^	4.23 ± 0.10 ^b^	2.94 ± 0.10 ^c^	7.45 ± 0.15 ^a^	4.14 ± 0.09 ^b^	3.29 ± 0.12 ^c^	56.7 ± 2.78 ^aB^	39.5 ± 2.39 ^bC^	55.7 ± 2.36 ^aB^	44.3 ± 2.52 ^bC^
	25	3.73 ± 0.09 ^a^	1.72 ± 0.09 ^b^	1.18 ± 0.14 ^c^	3.72 ± 0.08 ^a^	1.51 ± 0.07 ^bc^	1.32 ± 0.06 ^c^	46.3 ± 3.52 ^aC^	31.7 ± 4.49 ^bD^	40.5 ± 2.80 ^abC^	35.4 ± 2.42 ^bD^

Description as in Table 4.

**Table 7:** Extraction of basil phytochemicals.

Extraction Step	Solvent Ratio by Volume per 1 Part of Initial Plant Material (*v/w*)	Time (min)	Temperature (°C)
I	15	60	40
II	10	30	40
III	5	15	40

## Data Availability

The data presented in this study are available on request from the corresponding author.
